# Quercetin loaded polymeric dissolving microarray patches: fabrication, characterisation and evaluation

**DOI:** 10.1007/s13346-024-01616-8

**Published:** 2024-05-09

**Authors:** Qonita Kurnia Anjani, Natalia Moreno-Castellanos, Masoud Adhami, Delly Ramadon, Jangga Jangga, Ryan F. Donnelly

**Affiliations:** 1 School of Pharmacy, Medical Biology Centre, Queen’s University Belfast, 97 Lisburn Road, Belfast, Northern Ireland BT9 7BL UK; 2 Fakultas Farmasi, Universitas Megarezky, Jl. Antang Raya No. 43, Makassar, 90234 Indonesia; 3 Basic Science Department, Faculty of Health, Universidad Industrial de Santander, Bucaramanga, 680001 Colombia; 4 Faculty of Pharmacy, Universitas Indonesia, Depok, 16424 Indonesia

**Keywords:** Quercetin, Dissolving microarray patches, Cell proliferation, Inflammation activity

## Abstract

Quercetin, a natural compound, shows promising potential in wound healing by reducing fibrosis, limiting scar formation, and boosting fibroblast proliferation. However, its effectiveness is hindered by poor solubility, resulting in low bioavailability and necessitating high doses for therapeutic efficacy. This study presents a novel approach, fabricating quercetin-loaded microarray patches (MAPs) using widely employed solubility enhancement strategies. Fabricated MAPs exhibited favourable mechanical strength and could be inserted into excised porcine skin to a depth of 650 μm. Furthermore, formulations containing Soluplus^®^ significantly increased the drug loading capacity, achieving up to 2.5 mg per patch and complete dissolution within an hour of application on excised porcine skin. In vitro studies on full-thickness neonatal porcine skin demonstrated that Soluplus^®^-enhanced MAPs effectively delivered quercetin across various skin layers, achieving a delivery efficiency exceeding 80% over 24 h. Additionally, these prototype MAPs displayed anti-inflammatory properties and demonstrated biocompatibility with human keratinocyte skin cells. Therefore, quercetin-loaded MAPs employing Soluplus^®^ as a solubility enhancer present a promising alternative strategy for wound healing and anti-inflammatory therapy applications.

**Graphical abstract:**

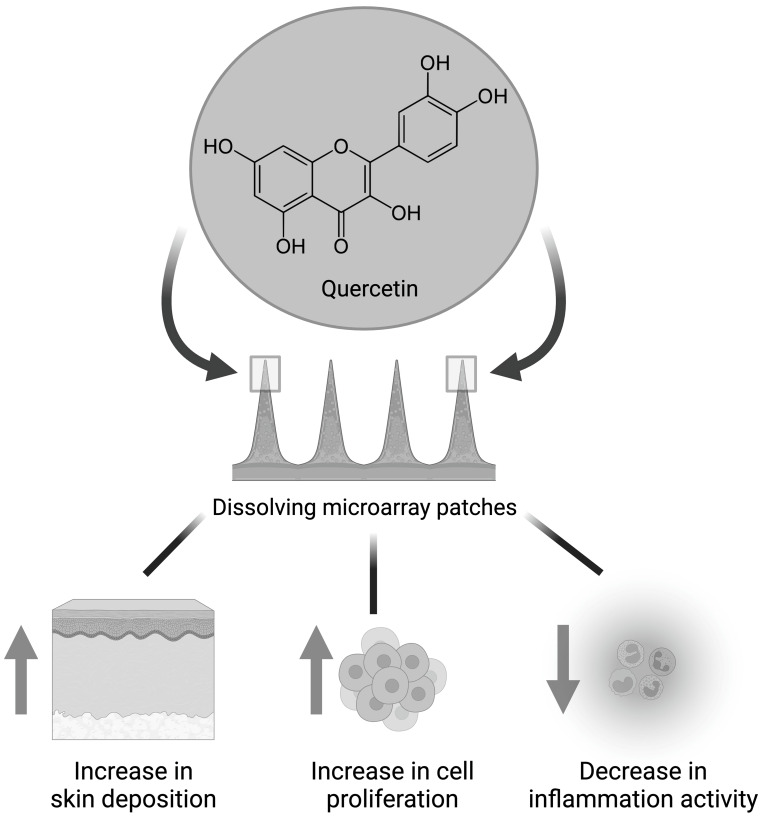

## Introduction

Quercetin, a polyhydroxy flavonoid found in various plant parts such as blossoms, leaves, and fruits, holds significant medical potential, due to its diverse pharmacological actions. These actions encompass anti-oxidative, anti-fibrotic, anti-cancer, and anti-inflammatory properties [1, 2]. Particularly noteworthy is quercetin’s ability to potentially enhance wound healing by reducing fibrosis, limiting scar formation, and promoting fibroblast proliferation [2]. Classified as a BCS class II drug, the efficacy of quercetin’s health benefits relies heavily on its absorption within the human body, which, in turn, hinges on its bioavailability in the digestive system [3]. However, being hydrophobic, quercetin exhibits limited solubility in water (0.17–7 µg/mL), gastric fluids (5.5 µg/mL), and small intestinal fluids (28.9 µg/mL) [4, 5]. This characteristic leads to precipitation in these fluids, diminishing its bioavailability.

To achieve an anti-inflammatory effect, two primary methods for drug administration are topical creams and hypodermic needles. While needles often cause discomfort, reducing patient acceptance, topical creams have lower bioavailability [6]. The skin, consisting of the *stratum corneum* in the epidermis, acts as the principal barrier to intradermal and transdermal drug delivery [7]. The selective permeability of the *stratum corneum*, especially to lipophilic and low molecular weight drugs, poses a significant challenge in designing effective topical formulations [8, 9]. Hence, researchers have focused on strategies to enhance intradermal delivery, where microarray patches (MAPs) emerge as a proactive approach.

MAPs, as minimally invasive devices, effectively bypass the primary skin barrier, facilitating localised drug delivery into the skin [10]. These patches create aqueous conduits and tiny holes upon skin application, enabling drugs or drug nanoparticles to penetrate deeper skin layer [11, 12]. The benefits of MAPs encompass painless delivery, rapid drug release, and improved patient compliance [13]. Additionally, MAPs, using self-dissolvable polymers in their production, mitigate the production of biohazardous waste [7]. For quercetin delivery *via* MAPs, Yi et al. fabricated soluble hydrogel MAPs consisting of GelMa in the first layer and PVA as the baseplate [14]. The researchers demonstrated that the MAP formulation was able to penetrate the skin and possessed good biocompatibility. Most importantly, the MAPs successfully showed wound healing activity both in vitro and in vivo settings. These MAPs were shown to promote collagen formation and neoangiogenesis as well as reduce oxidative stress levels. This result suggests that MAPs could be a potential platform to deliver quercetin for local delivery, specifically for wound healing treatment. However, despite these potentials, the work presented by Yi et al. did not include any drug content and ex vivo release or deposition studies, which still need exploration to evaluate the choice of polymer to maximise drug loading and then reduce the patch size as a result. Therefore, there is an impetus to explore solubility enhancement in MAP fabrication for quercetin to overcome this issue.

This present study utilises quercetin to demonstrate how MAPs, coupled with solubility enhancement strategies (using Soluplus^®^), can markedly improve transdermal drug delivery, particularly for poorly soluble drugs. We have previously reported that Soluplus^®^ (polyvinyl caprolactam-polyvinyl acetate-polyethylene glycol grafted copolymer) could increase the solubility and permeability of colchicine by preparing the tips of needles of active substances and Soluplus^®^*via* the solid dispersion technique [15]. However, in this present study, we prepared the MAPs using a different approach by involving Soluplus^®^ in the combination of PVA and PVP *via* the double casting technique. The fabricated MAPs underwent assessment for mechanical resistance, insertion capabilities, and skin deposition. Additionally, the anti-inflammatory properties of quercetin loaded into the MAPs were evaluated in an in vitro study conducted on keratinocyte cells. This innovative approach holds considerable promise as an alternative for the management of wound healing and inflammatory reactions.

## Materials and methods

### Materials

Quercetin (with a purity of ≥ 95%), Dulbecco’s Modified Eagle’s medium (DMEM), fetal bovine serum (FBS), and the 3-(4,5-dimethylthiazol-2-yl)-2,5-diphenyltetrazolium bromide (MTT) assay kit, along with dimethyl sulfoxide (DMSO), were sourced from Sigma Aldrich (St. Louis, MO, USA). The human epidermal keratinocytes (HaCaT) cell line was acquired from ATCC. Poly(vinyl alcohol) (PVA, with a molecular weight of 10,000 g/mol) was obtained from Sigma-Aldrich (Dorset, UK). Soluplus^®^ was generously provided by BASF SE (Ludwigshafen, Germany). Plasdone™ K-29/32 (PVP, with a molecular weight of 58,000 g/mol) and Plasdone™ K-90 (PVP, with a molecular weight of 1,300,000 g/mol) were purchased from Ashland Industries Europe GmbH (Schaffhausen, Switzerland). Glycerol was sourced from VWR (Leicestershire, UK). Phosphate-buffered saline tablets (PBS, with a pH range of 7.3–7.5) were obtained from Sigma-Aldrich (Dorset, UK). The enzyme-linked immunosorbent assay (ELISA) analysis kit was procured from R&D Systems (Minneapolis, MN, USA). All solvents utilised were of analytical grade and obtained from Sigma-Aldrich (Dorset, UK).

### Determination of wetting angle of quercetin

The wetting angle between quercetin and a Soluplus^®^ surfactant solution was measured employing an Attension Theta optical tensiometer (Biolin Scientific, Gothenburg, Sweden) following a methodology akin to previously published studies [16, 17]. Initially, 50 mg of quercetin was accurately weighed and compressed using 4 tonnes pressure into a tablet to ensure a flat surface [18]. The sessile drop method was then utilised to determine the wetting or contact angle between the quercetin tablet and a Soluplus^®^ solution of 0.5% w/v concentration, with deionized water serving as the control. A 4 µL droplet of each Soluplus^®^ solution (0.5% w/v) or water was dispensed onto the surface of the quercetin tablet, and the wetting angle was measured precisely 30 s after droplet deposition. The collected data were subsequently analysed using OneAttension software for comprehensive evaluation and interpretation.

### Fabrication of quercetin loaded dissolving MAPs

A double casting method was employed to fabricate two-layered dissolving MAPs loaded with quercetin. The first layer, which forms the tips of the MAPs, consisted of quercetin and an aqueous solution of PVA-PVP (PP2) in a 1:1 ratio at a concentration of 40% w/w each. Two different formulations were tested to achieve a first layer with appropriate mechanical properties, as detailed in Table 1.

**Table 1:** Formulations of the first layer of dissolving MAPs

Materials	Compositon (mg)	
	F1	F2
Quercetin	15	15
PP2	20	20
Deionised water	65	-
Soluplus^®^ solution (0.5% w/v)	-	65

Quercetin was incorporated into the polymeric mixture using a SpeedMixerTM DAC 150.1 FVZ-K (GermanEngineering, Hauschild & Co. KG, Hamm, Germany) operating at 3,500 rpm for 5 min. The resulting blend was poured into a 16 × 16 silicone mould designed with pyramidal needles measuring 850 μm in height, 300 μm in base width, 300 μm interspacing, and covering a 0.36 cm^2^ patch area. The filled mould was then placed in a positive pressure chamber (Protima^®^, TÜV Rheinlad, Koln, Germany) set at 5 bar pressure for 5 min to ensure complete filling of the needle cavities. Afterward, any excess product on the mould surface was meticulously removed, and the first layer-filled mould underwent another round of positive pressure treatment at 5 bars for 30 min. Elastic-based flexible rings were affixed to the top of the mould using a 40% w/w PVA aqueous solution (MW 9–10 kDa), following established procedures [19–22].

Subsequently, the first layer-filled mould was left to dry at ambient conditions overnight to allow for water evaporation. The following day, the baseplate, composed of a 30% w/w PVP (MW 90 kDa) and 1.5% w/w glycerol aqueous blend, was cast as the second layer. To ensure optimal distribution and removal of air gaps, the moulds underwent centrifugation at 3,500 rpm for 10 min before being left to dry overnight under ambient conditions. Once fully dried, the MAPs were carefully removed from the moulds, and any excess baseplate side walls were precisely trimmed using scissors. The formed MAPs were further dried at 37 °C for 24 h before undergoing characterisation.

### Evaluation of mechanical properties and insertion profile of dissolving MAPs

The Leica digital light microscope (Leica EZ4 D, Leica Microsystems, Milton Keynes, UK) was employed to examine the morphology of MAPs (formulations F1 and F2) and estimate the mean needle height. Compressive resistance analysis of the formulation was conducted using a TA-TX2 Texture Analyser (TA) (Stable Microsystems, Haslemere, UK), following established protocols [23–25]. A compression force of 32 N was applied for 30 s at a rate of 0.5 mm/sec to simulate the pressure exerted by a human thumb during patch application (*n* = 25). Evaluation of the compressive resistance of MAPs involved measuring the reduction in needle height post-compression, calculated using Eq. 1.

1 $$ Variation\;in\;needle\;height \left(\%\right)= \frac{\varDelta\;needle\;height}{initial\;needle\;height } \times 100$$

Where Δ represents the difference in needle height before and after the compression test.

The efficacy of MAPs to penetrate the skin was evaluated through both in vitro and ex vivo experiments. In the in vitro study, eight layers of Parafilm^®^ M were stacked, and MAPs (F1 and F2) were inserted into each layer. The depth of insertion was observed using a digital light microscope. In the ex vivo experiment, neonatal porcine skin was employed to validate the performance of MAPs. The insertion of MAPs was monitored in real-time using an EX-101 optical coherence tomography (OCT) microscope (Michelson Diagnostics Ltd., Kent, UK), and the depth of insertion was analysed using ImageJ^®^ software (National Institutes of Health, Bethesda MD, USA). The porcine skin utilised in the experiment was obtained from stillborn piglets within 24 h post-mortem and was stored at -20 °C until utilised.

### Evaluation of drug content of dissolving MAPs

The drug content of dissolving MAPs was quantified by dissolving the MAP in 4 mL of deionised water to solubilise the polymer matrix, followed by sonication for 30 min using a bath sonicator. Once the MAPs were completely dissolved, 4 mL of methanol was added to solubilise the quercetin, followed by another sonication cycle for 30 min. This mixture was then centrifuged at 14,500 rpm for 15 min, and the supernatant was collected and analysed using HPLC. Three samples per each formulation were evaluated to obtain the results (*n* = 3).

To calculate the theoretical drug content localised in the needle tips, Eq. 2 was used as follows [13]:

2 $$ Theoritical\;drug\;content =N\times \frac{\left(h.{a}^{2}\right).\rho.\left[drug\right]}{3}$$

Where N is the number of needle tips per patch, h is the height of the tips containing drug (mm), a^2^ is the width of the needle area (mm), ρ is the density of the dry formulation (mass/volume), and [drug] is the concentration of drugs in the dry formulation (mg drug/mg total formulation of the first layer).

### In situ skin dissolution studies

The study aimed to explore the in situ skin dissolution process by evaluating the duration required for the MAPs to dissolve ex vivo in neonatal porcine skin. Full-thickness neonatal porcine skin tissue was prepared by soaking it in PBS (pH 7.4) for 30 min at 37 °C until reaching equilibrium. The MAPs were manually inserted into the skin using thumb pressure for 30 s, and to prevent displacement, a cylindrical stainless steel metal weight weighing approximately 15 g was placed atop the MAP. Subsequently, the samples were placed in an oven (Genlab incubator, Genlab Ltd., Cheshire, UK) set at 37 °C and carefully extracted from the skin at 5, 15, or 30 min intervals. Finally, the MAPs were observed under a digital microscope.

### Ex vivo deposition studies

The delivery efficiency of quercetin to various layers of full-thickness neonatal porcine skin was evaluated using a Franz cell diffusion setup (PermeGear, Inc., Hellertown, PA, USA). Initially, the full-thickness neonatal porcine skin was appropriately sized and affixed to the donor compartment of the Franz cells, with the subcutaneous side facing the receiver compartment, using cyanoacrylate glue (Stick it^®^ super glue, PLDZ Pattison House, Dublin, ROI). Subsequently, the manually inserted MAPs were pressed onto the skin (*stratum corneum* side) using thumb pressure for 30 s. The receiver compartment of the Franz cells was filled with PBS (pH 7.4) as the receiver medium and maintained at 37 °C using a thermal water jacket to mimic physiological conditions. The medium was continuously stirred at 600 rpm with metal bars. To prevent the expulsion of MAPs from the skin during the experiment, a cylindrical stainless steel weight (approximately 15 g) was placed on top of the MAPs, and a metal clamp was used to secure the donor and receiver compartments. Skin and receiver compartment samples were collected at the 24-hour mark. Samples from the receiver compartment were centrifuged at 15,300 rpm for 15 min, filtered through a 0.45 μm PTFE membrane filter, and analysed for quercetin content using HPLC. Skin samples were also collected and processed to separate the epidermis and dermis layers similar to previously published studies [17, 21]. The epidermis layer was homogenized with 2 mL of methanol using a thermal mixer (ThermoMixer F2.0, Eppendorf, Hamburg, Germany) to extract quercetin. Similarly, the dermal layer was homogenised in 0.5 mL of deionised water using Tissue Lyser LT (Qiagen Ltd., Manchester, UK) at 50 Hz for 15 min, followed by the addition of 1 mL of methanol and homogenization. The supernatant obtained after centrifugation at 15,300 rpm for 15 min was used for HPLC analysis.

### Cytocompatibility studies

To assess the effect of MAPs on epidermal keratinocyte cells, we evaluated the biocompatibility of MAPs containing quercetin, and blank MAPs. Human epidermal keratinocytes were cultured in DMEM supplemented with 10% (v/v) heat-inactivated fetal bovine serum and 1% penicillin/streptomycin. The cell cultures were maintained at 37 °C in a humidified 5% CO_2_ incubator. Upon reaching confluence, keratinocytes were seeded at a density of 5 × 10^4^ cells on 24-well plates and cultured for 72 h. To assess the effect of MAPs on cell viability, we employed the MTT assay, following a procedure similar to that described by Dominguez-Robles et al. [26]. Briefly, after 72 h, MTT reagent was added to each sample, and after a 6-hour incubation, DMSO was used as a dissolution reagent. Absorbance was recorded at 540 nm using a spectrophotometer. Furthermore, a LIVE/DEAD cytotoxicity assay was conducted on keratinocytes treated with MAPs samples. Cell toxicity was observed using a fluorescence microscope at 100× magnification after treatment with a mixture of 2 mM acetomethoxy derivate of calcein (calcein-AM) and 4 mM ethidium homodimer-1 (EthD-1) at 37 °C for 30 min. The cell proliferation of human keratinocytes was evaluated by measuring the number of DNA copies using a picogreen assay, as previously described [27]. Briefly, DNA samples were extracted, and a DNA-binding fluorescent dye solution was used to measure fluorescence intensity at an excitation wavelength of 480 nm and an emission wavelength of 520 nm. Lambda DNA was employed to create a standard curve for calculating DNA amounts. All biocompatibility studies were conducted in triplicate for accurate assessment.

### In vitro anti-inflammation activity

To assess the impact of MAPs loaded with quercetin and blank MAPs on the release of IL1-β, IL-8, and TNF-α, human epidermal keratinocytes were cultured at 37 °C in a 5% CO_2_ and 95% air humidified environment. Upon reaching confluence, the keratinocytes were subjected to lipopolysaccharide (LPS) treatment, an accepted pro-inflammatory model, known for its induction of inflammation in vitro. LPS was introduced to each experimental condition at a concentration of 2.5 µg/mL for an additional 4 h. After treatment, the supernatants were collected and transferred into sterile 96-well plates for ELISA analysis. Kits were utilised to measure the production of IL-6, IFN-γ, and TNF-α, following the manufacturer’s guidelines. To account for potential variations in cell numbers, the release of IL-6, IFN-γ, and TNF-α into the medium was normalised to total protein content.

### High-performance liquid chromatography (HPLC) analysis

The amount of quercetin was quantified using reversed-phase high-performance liquid chromatography (HPLC) with an Agilent Technologies 1220 Infinity compacted LC series system (Agilent Technologies UK Ltd, Stockport, UK) equipped with a UV detector. Chromatographic separation was performed on an XSelect CSH C18 column with dimensions of 3.0 mm internal diameter, 150 mm length, 3.5 μm particle size, and a pore size of 130 Å (Waters, Dublin, Ireland). Prior to the column, a VanGuard^®^ cartridge (3.9 mm internal diameter, 5 mm length) with chemistry similar to the main column was installed. The sample elution was achieved using a mobile phase consisting of 0.1% v/v trifluoroacetic acid and acetonitrile (50:50 v/v) at a flow rate of 0.6 mL/min. HPLC analysis was carried out at 30 °C with a 10 µL injection volume over a 10-minute duration, and detection was performed at a wavelength of 378 nm.

### Statistical analysis

The data analysis and interpretation were performed using GraphPad Prism^®^ version 9.4 (GraphPad Software, San Diego, California, USA). To compare multiple groups, we employed one-way analysis of variance (ANOVA), with a significance level set at *p* < 0.05. Unless explicitly stated otherwise, the data are presented as means ± standard deviation (SD).

## Results and discussion

### Determination of wetting angle of quercetin

**Fig. 1:**
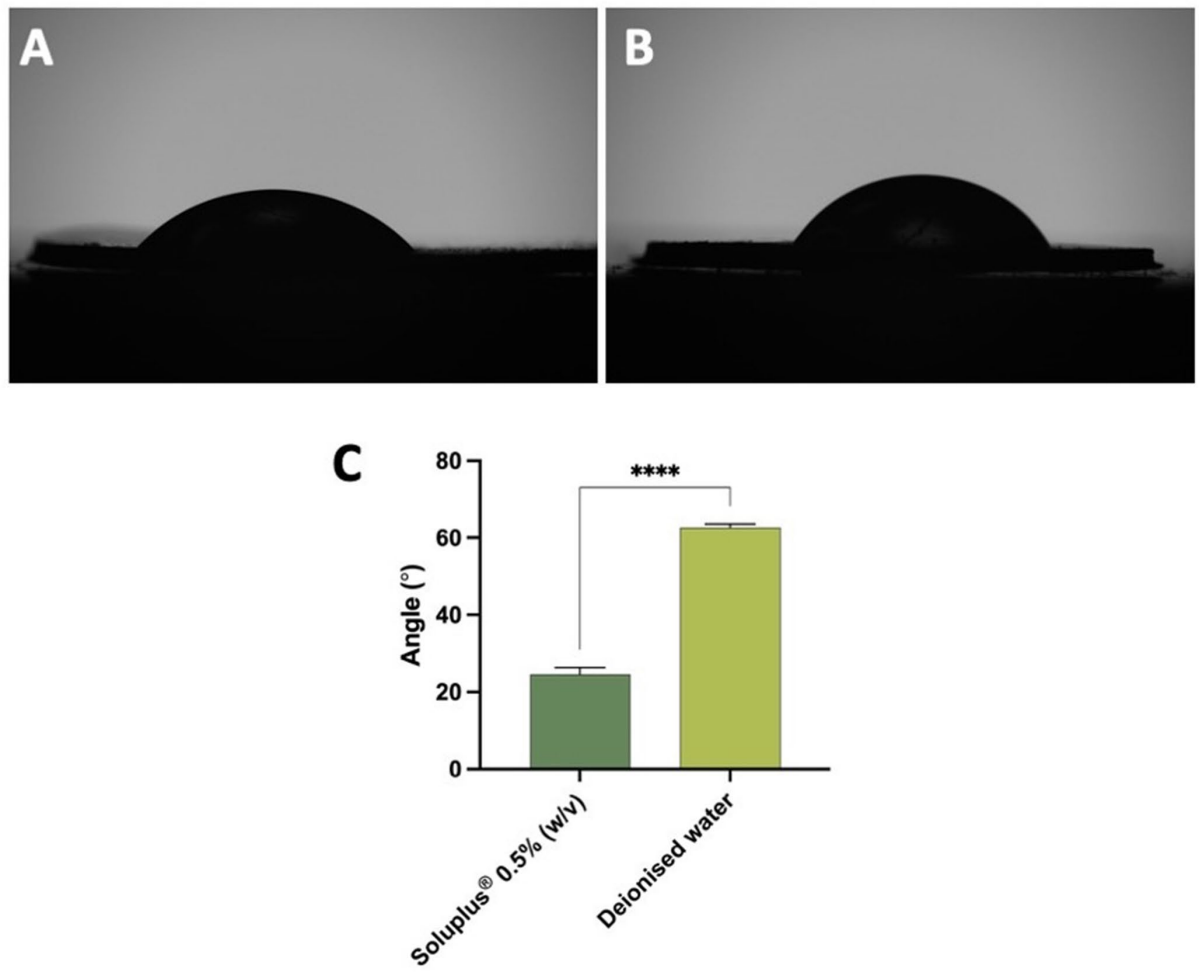
Representative images for wetting angle measurement for (**A**) Soluplus^®^ 0.5% (w/v) and (**B**) deionised water. (**C**) Wetting angle measurement for quercetin with Soluplus^®^ 0.5% (w/v) and deionised water (means + SD, *n* = 3)

To assess the hydrophilicity of quercetin concerning Soluplus^®^ and deionised water, the wetting angle was measured, as depicted in Fig. 1. Figure 1C illustrates that the wetting angle of Soluplus^®^ 0.5% (w/v) was significantly lower than that of deionised water (*p* < 0.05). This suggests that Soluplus^®^ effectively reduces surface tension, resulting in a significant decreased wetting or contact angle (*p* < 0.05). This finding aligns with our prior research, demonstrating that the surfactant solution lowered contact angles between both hydrophilic and hydrophobic drugs in comparison to the control group (deionised water) [16]. Quercetin, classified as a BCS class II drug [3], exhibits high permeability but low solubility. Improved solubility could enhance the drug’s efficacy and delivery potential. Given that the contact angle of quercetin with Soluplus^®^ is 2.5-fold lower than that in deionised water, it suggests that the Soluplus^®^ solution tends to spread across the surface of quercetin, forming a flatter droplet and thereby reducing the contact angle [28, 29]. This result indicates that surfactant could help quercetin to disperse better in the formulation, which could lead to higher drug content and faster dissolution of tips of MAPs [16]. Therefore, this strategy, by using solubility enhancement, could contribute to better drug delivery outcomes in the skin.

### Fabrication and characterisation of quercetin loaded dissolving MAPs

**Fig. 2:**
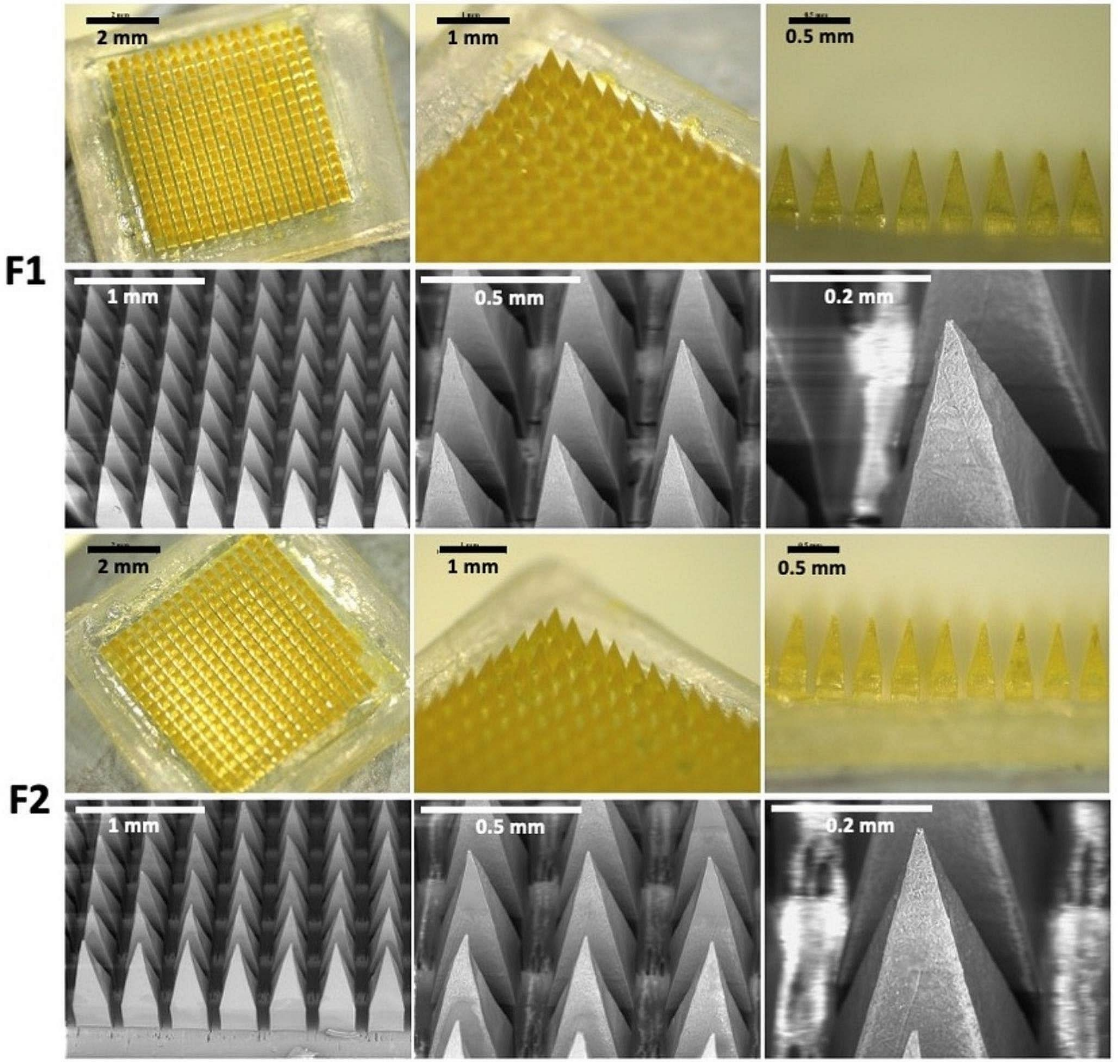
Digital and SEM images of dissolving MAP loaded with quercetin. F1: MAP formulation prepared from aqueous solution containing quercetin 15% w/w, PP2 20% w/w, and deionised water 65% w/w. F2: MAP formulation prepared from aqueous solution containing quercetin 15% w/w, PP2 20% w/w, and Soluplus^®^ solution (0.5% w/v) 65% w/w

After measuring the wetting angle of quercetin to Soluplus^®^ and deionised water, dissolving MAP formulations containing surfactant was fabricated using the double casting technique, as previously detailed [17, 25]. Figure 2 displays the resulting MAPs, exhibiting uniform arrays of microneedles on a smooth, flat baseplate. Notably, there were no observed air bubbles or unevenly formed needles. The physical appearance of the quercetin-loaded MAPs showed yellowish tips, indicating successful concentration and localization of quercetin at the tips rather than dispersion in the baseplate. This concentration at the tips holds potential to enhance delivery efficiency and reduce drug wastage.

**Fig. 3:**
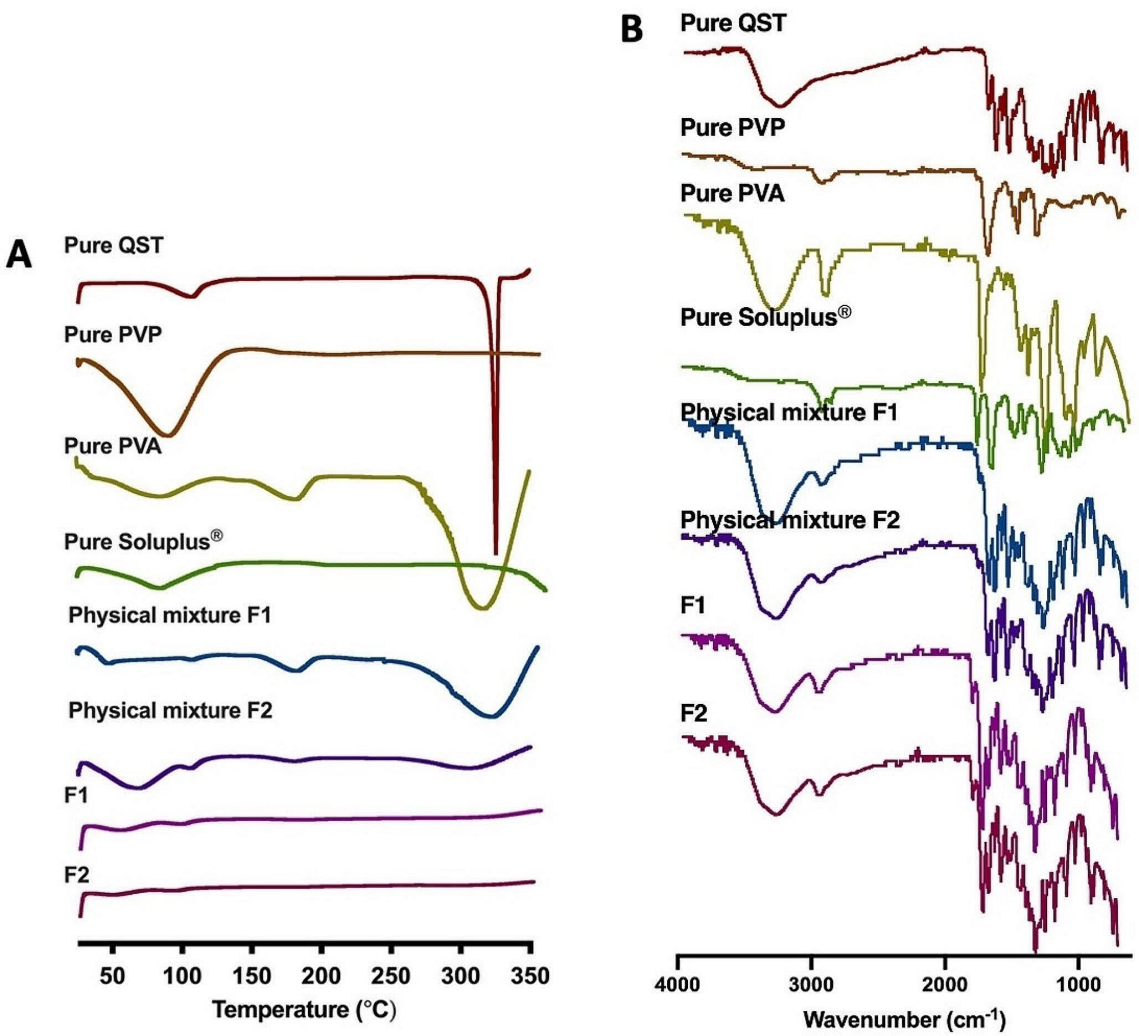
(**A**) DSC and (**B**) FTIR profile of pure quercetin, pure PVP, pure PVA, pure Soluplus^®^ physical mixture F1, Physical mixture F2, MAP F1 and MAP F2

The properties of quercetin in the solid state, post its loading into MAPs, were assessed using DSC and FTIR analyses. This physiochemical characterisation is crucial to monitor during MAP preparation, ensuring the uniformity of the produced MAPs and complete encapsulation of quercetin into the polymeric matrix in the first layer. Figure 3A and B illustrate the DSC thermograms and FTIR spectra, respectively. DSC analysis (Fig. 3A) aimed to examine the physicochemical interactions between quercetin, surfactant, and polymers. It also sought to discern any changes in crystallinity following the MAP fabrication step. The thermogram of pure quercetin displayed a sharp endothermic peak at 324 °C, indicating its melting point. Additionally, pure PVP, PVA, and Soluplus^®^ exhibited several broad endothermic peaks at 89 °C, 314 °C, and 75 °C, respectively, representing their respective excipient melting points in the MAP formulations. However, the DSC thermograms of F1 and F2 did not exhibit any endothermic peaks post-MAP fabrication. This suggests the successful incorporation of quercetin within the polymers, with the drug being present in a state of low crystallinity in the MAP formulations.

The FTIR analysis (Fig. 3B) depicted distinctive spectra for pure quercetin, PVP, PVA, Soluplus^®^, physical mixture, and MAP formulations. The spectrum of pure quercetin displayed O-H stretching at 3310 cm^− 1^ and a C = O group at 1610 cm^− 1^. Both pure PVP and PVA showed C-H stretching at 2980 cm^− 1^, and pure PVA exhibited an additional O-H stretching band at 3350 cm^− 1^. Soluplus^®^ displayed peaks at 1477 cm^− 1^, 1635 cm^− 1^, and 1734 cm^− 1^, indicating C-O-C stretching, C = O groups, and C = O ester groups, respectively. Comparing the spectra of pure quercetin and MAP formulations (F1 and F2) revealed similar patterns, with a minor peak at 2980 cm^− 1^ in the MAPs. This suggests that MAP fabrication did not induce significant structural changes in the drug. These findings align with the DSC analysis, confirming successful encapsulation of the drug within the PVA and PVP polymer structures.

### Evaluation of mechanical properties

**Fig. 4:**
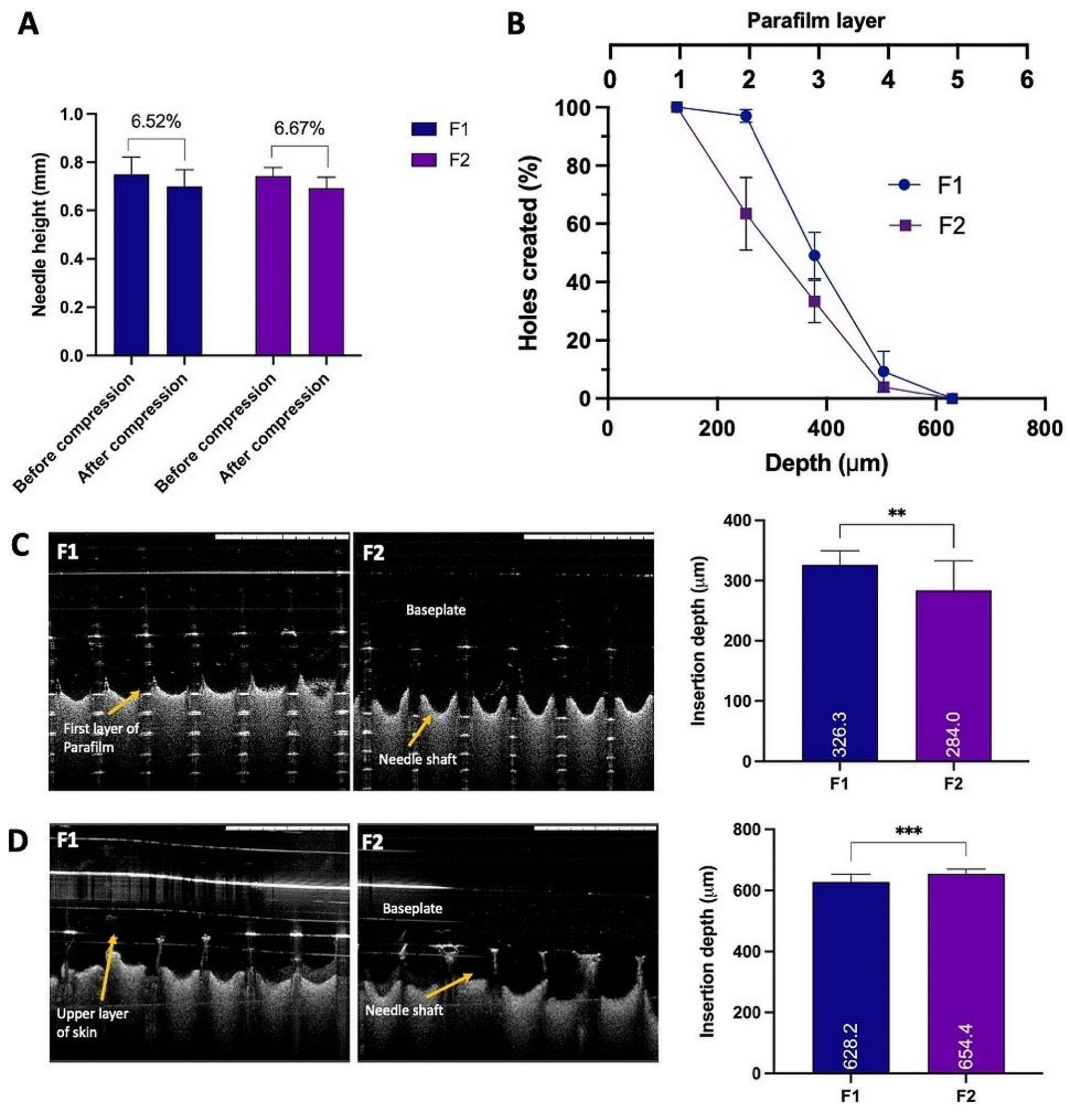
(**A**) MAPs height reduction for needles loaded with quercetin following the application of a 32 N compressive force (means + SD, *n* = 20). (**B**) Percentage of channels formed per Parafilm^®^ M layer upon the application of quercetin loaded dissolving MAPs (means ± SD, *n* = 3). Insertion depth of quercetin loaded dissolving MAPs into (**C**) Parafilm^®^ M and (**D**) full thickness ex vivo neonatal porcine skin monitored in situ using optical coherence tomography (OCT) (means + SD, *n* = 20). Scale bar = 1 mm

The mechanical properties of the MAPs were assessed post-fabrication. Figure 4A illustrates that both formulations, F1 and F2, experienced a reduction in needle height of less than 10% during a compression test employing a force of 32 N, equivalent to the thumb pressure typically applied during MAP insertion [30]. Notably, the results displayed no significant differences in needle height before and after compression for either formulation (*p* > 0.05). However, these findings contradict our earlier research involving surfactant incorporation into MAP formulations [16]. Previous studies indicated that surfactants could enhance needle mechanical resistance due to molecular interactions with the polymer matrix within the MAPs. Yet, it is essential to recognise that Soluplus^®^, employed in the current formulation, differs in properties from other surfactants like Pluronic^®^ F88, Lutrol^®^ F108, and Tween^®^ 80 utilised previously. Soluplus^®^ initially exists in a glassy and brittle state, potentially causing fragility and challenging removal from the mould after the casting process [31, 32]. However, in the current setup, the initial layer of MAPs comprises Soluplus^®^, PVA, PVP, and quercetin, possibly counteracting the Soluplus^®^ brittleness, resulting in comparable properties with the other formulation lacking Soluplus^®^. Moreover, there was no significant difference in the percentage reduction of needle height between F1 and F2 (*p* > 0.05). This outcome was anticipated since the MAPs prepared had similar needle dimensions, with no significant variation in height and diameter, as the moulds used were identical. In this study, a compression force of 32 N was applied, equivalent to the pressure exerted by a human thumb during MAP application [30]. However, it is important to consider the potential consequences of excessive force during insertion, as reported by Ando et al., where needle deformation leading to buckling and unbuckling can be predicted based on the needle aspect ratio (needle height/base diameter) [33]. With a needle aspect ratio of 2.8, buckling may occur with excessive force application, whereas with a ratio of 1.8, unbuckling may occur. Given that the dimensions of the MAPs in this study are 850 μm and 300 μm for the needle height and base diameter, respectively, buckling may occur as the mechanical failure mechanism if excessive force is applied during application. Since this ratio is crucial for predicting the effect of needle aspect ratio on needle fracture force, it is imperative to consider these parameters seriously during formulation development. Furthermore, the observed reduction in height for both fabricated MAP formulations remained below 15%, closely aligning with values reported for dissolving MAPs in prior studies [34–37]. Thus, these results indicate that the MAPs in this study exhibit the mechanical robustness necessary to withstand the compression forces likely exerted during MAP application.

Figure 4B demonstrates the penetration profile of MAPs into eight layers of Parafilm^®^, a validated skin simulant utilized in insertion studies to assess needle penetration capabilities [30, 38]. Both formulations (F1 and F2) successfully breached the initial layer of Parafilm^®^ (~ 126 μm thickness) at a force of 32 N. Subsequently, approximately 60% of F2 needles penetrated the second layer, whereas F1 achieved 100% penetration in the same layer. This difference might relate to the mechanical properties of Soluplus^®^ in F2, which exhibits some brittleness compared to the control formulation (F1). However, there were no significant differences between F1 and F2 in the penetration profiles upon reaching the third and fourth layers (*p* > 0.05). Notably, no observable penetration was achieved beyond the fifth layer.

When visualising the insertion of MAPs into Parafilm^®^ using OCT, both MAP formulations (F1 and F2) were observed to reach the fourth layer, as depicted in Fig. 4C. This resulted in insertion depths of approximately 326 μm for F1 and 284 μm for F2. The significant difference in Parafilm^®^ insertion depth between F1 and F2 (*p* < 0.05) aligns with earlier results from insertion profile studies, indicating that F2, due to its brittle nature, generated fewer holes, particularly in the second Parafilm^®^ layer. However, upon evaluating skin insertion in excised full-thickness neonatal porcine skin, both MAP formulations (F1 and F2) achieved significantly greater insertion depths (*p* < 0.05) compared to those in Parafilm^®^, as shown in Fig. 4D. This discrepancy may be attributed to Parafilm^®^’s nature derived from paraffin wax, which inadequately represents the properties of actual skin [30]. Additionally, as the MAPs consist of hydrophilic polymers and even though the drug is classified as BCS class II, it is completely encapsulated within the polymer matrix. The excised full-thickness neonatal porcine skin containing interstitial fluid, rich in moisture, might facilitate lubrication and promote needle insertion into deeper layers compared to the dry, moisture-lacking Parafilm^®^ [16]. This observation is further supported by the results depicted in Fig. 4D, demonstrating that F2 exhibited significantly deeper insertion into the excised full-thickness porcine skin (*p* < 0.05). This could be attributed to the incorporation of Soluplus^®^ in the MAP formulation, enhancing hydrophilicity and facilitating insertion into ex vivo skin. In summary, the disparity in insertion depth between Parafilm^®^ and excised full-thickness porcine skin aligns with prior evaluations of MAP insertion profiles [18, 34, 37–41]. However, despite all the differences between these two models, Parafilm^®^ has been validated as an alternative for insertion study of MAP platform, which can be used for comparative formulation studies [42]. Parafilm^®^ has been demonstrated and validated that it can be used as an alternative of excised porcine skin for insertion studies. In the previous study, Larraneta et al. has shown that the force that generated to apply MAPs by human volunteer was in the average of 20–40 N for 30 s [30]. Gantrez^®^ S-97 and PEG based MAPs were used in the study which contain no drugs, therefore there is no discrepancy was observed between the Parafilm and excised skin during the insertion study. However, the result from insertion studies of the current work is aligned with our previous studies [18, 33, 36–39] where the dissolving MAPs containing the active compounds and the insertion in Parafilm^®^ is significantly lower compared to excised porcine skin. Overall, the Parafilm^®^ can be used as a skin simulant for MAP insertion evaluation as long as the force applied during insertion is in the range of 20–40 N for 30 s. This condition is equal to the force that generated by human thumb and still in the acceptable range of force to avoid buckling or any deformation due to excessive force application.

### Evaluation of drug content of dissolving MAPs

**Fig. 5:**
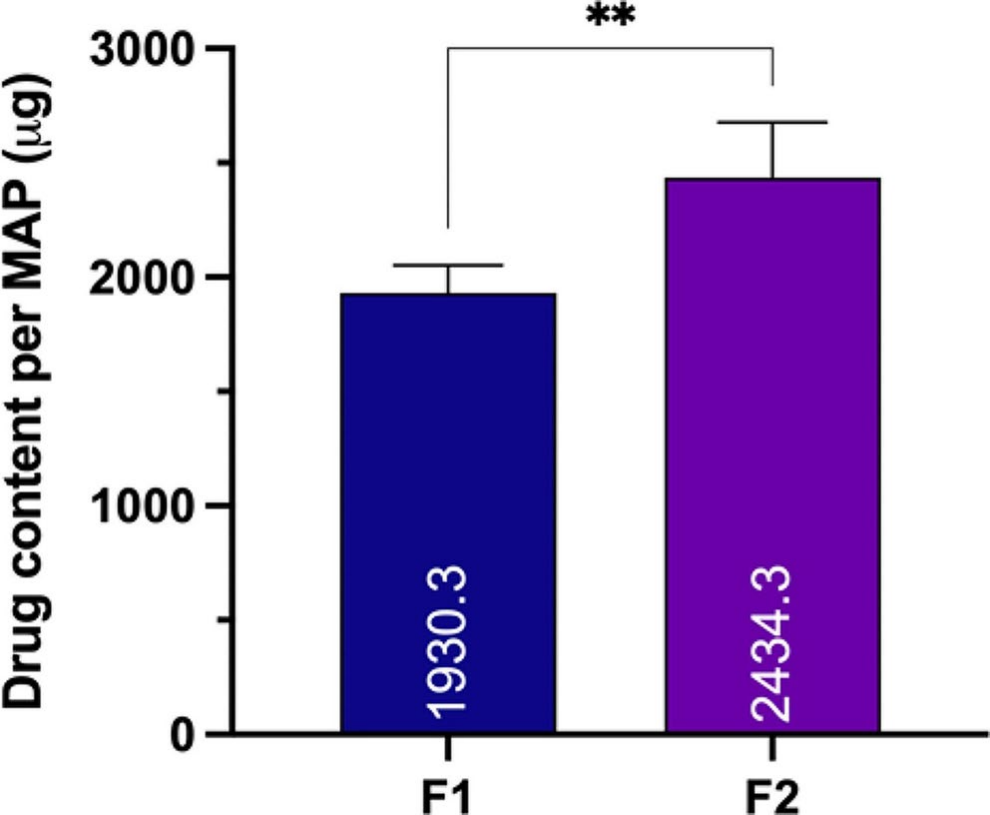
Drug content of quercetin loaded dissolving MAPs (means + SD., *n* = 3)

In relation to drug content, Fig. 5 illustrates a significant impact (*p* < 0.05) on the quantity of drugs that can be loaded into the MAPs due to the incorporation of Soluplus^®^ as a surfactant within the formulation. Previously mentioned, quercetin falls under BCS Class II, characterised by low permeability and solubility [43]. Incorporating Soluplus^®^ is known to enhance the solubility of quercetin [32], thereby enabling a greater quantity of the drug to be loaded into the needle tips of the MAPs. These values were equal to 55% and 69% of drug content observed in F1 and F2, respectively, compared to the theoretical drug content in the needle tips (approximately 3.51 mg for both F1 and F2). These discrepancies might be affected by the manual removal of excess formulation during the preparation of dissolving MAPs.

### In situ skin dissolution studies

**Fig. 6:**
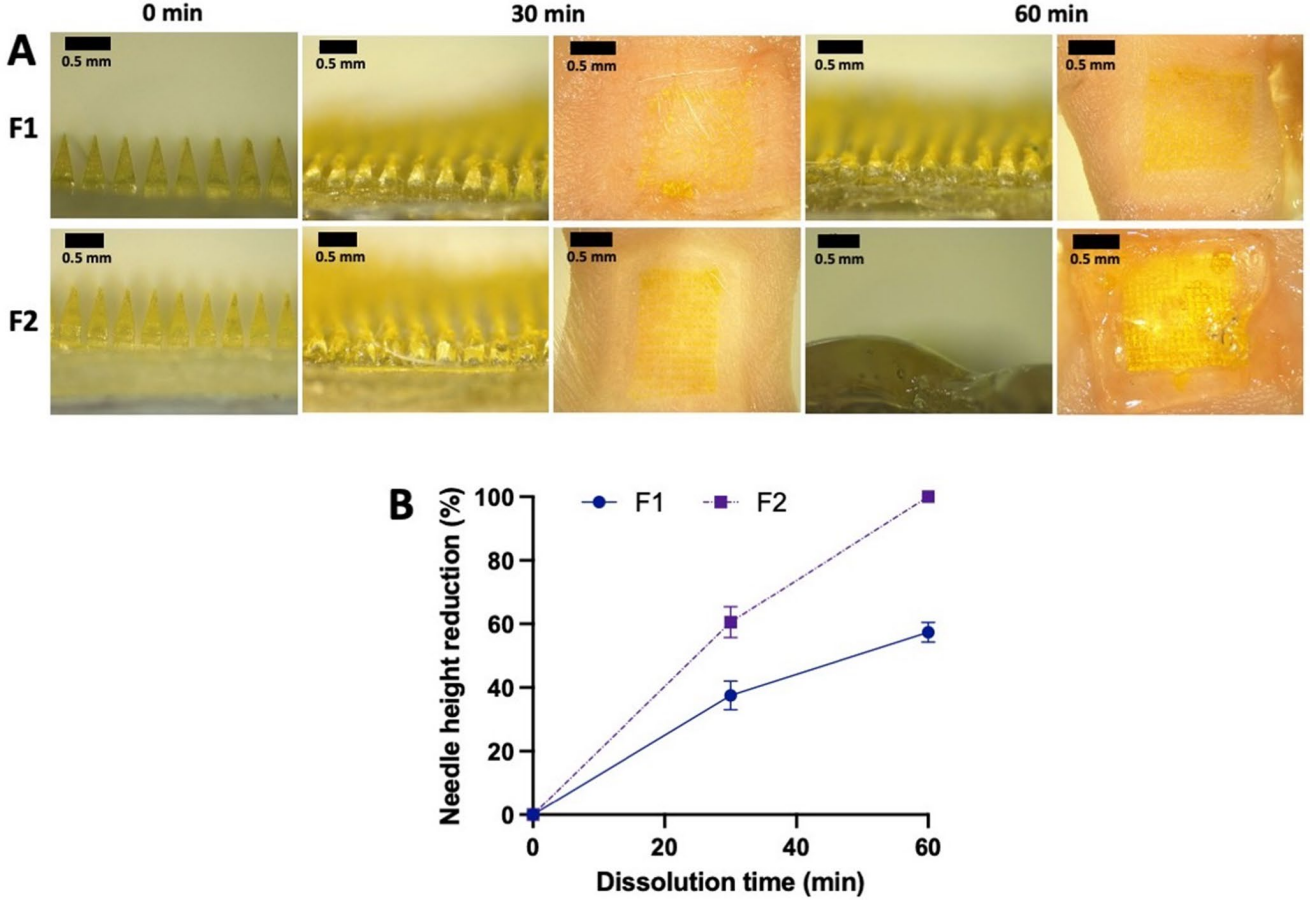
(**A**) Digital images of needle dissolution at 0, 30 and 60 min, following insertion into and removal from excised neonatal porcine skin ex vivo. (**B**) Profile of needle height reduction (%) during the in situ skin dissolution studies (means ± SD, *n* = 10). F1: MAP formulation prepared from aqueous solution containing quercetin 15% w/w, PP2 20% w/w, and deionised water 65% w/w. F2: MAP formulation prepared from aqueous solution containing quercetin 15% w/w, PP2 20% w/w, and Soluplus^®^ solution (0.5% w/v) 65% w/w

The in situ dissolution studies aimed to determine the timeframe and process involved in the complete dissolution of MAPs upon skin insertion. As depicted in Fig. 6A, after a 1-hour application, the needle layer of F2 MAP underwent full dissolution, transferring all drug content into the skin. However, only 57% of needle tips were dissolved after 1-hour for F1 (without any surfactants) (Fig. 6B). Furthermore, upon leaving F2 MAP on the skin for an hour, complete dissolution of the entire patch, including the polymeric baseplate, was observed, resulting in an occlusive and adhesive polymer gel forming on the skin surface. In contrast, F1 MAP required a longer duration to achieve complete dissolution and deposit its content into the skin. The addition of Soluplus^®^ in the formulation of F2 demonstrated enhanced solubility of quercetin loaded in the MAP, contributing to improved dissolution of the entire patch, as previously reported [16, 32].

### Ex vivo deposition studies

**Fig. 7:**
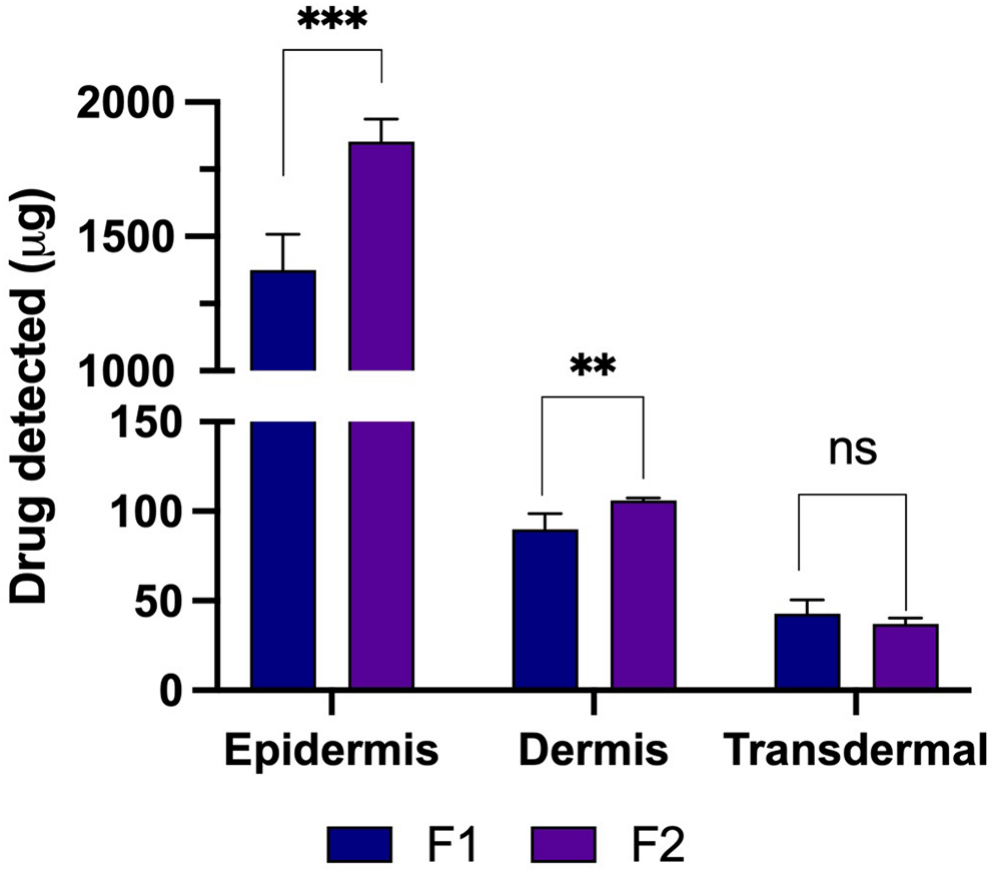
Amounts of quercetin extracted from different skin layers: epidermis and dermis from excised neonatal porcine skin as well as quercetin delivered transdermally at 24 h in an in vitro Franz cell diffusion study (means + SD, *n* = 5)

The ex vivo skin deposition studies using full-thickness neonatal porcine skin were conducted, and the findings are presented in Fig. 7. The assessment of quercetin delivery across different skin layers revealed a significantly higher amount of drug delivered from formulation F2 compared to F1 within the epidermis layer (*p* < 0.05). Specifically, approximately 1850 µg (~ 76%) from F2 and approximately 1500 µg (~ 77%) from F1 were observed, suggesting an accumulation of quercetin within this layer, forming a secondary drug reservoir. This reservoir potentially facilitates a slow release of the drug into the deeper dermis layers over time, beneficial for its anti-inflammatory effects, as quercetin’s primary site of action is predominantly localised in the epidermis layer [44–47]. Moreover, in the dermis layer, F2 exhibited significantly higher drug delivery (*p* < 0.05) with approximately 106 µg (~ 4.3%) compared to 94 µg (~ 4.9%) for F1. However, no significant difference was found between F1 and F2 in transdermal delivery over 24 h (*p* > 0.05). Consequently, both MAP formulations showed an overall delivery efficiency of more than 80% within 24 h.

Regarding quercetin delivery *via* MAP, Paleco et al. developed a silicon-based MAP combined with lipid microparticles [44]. Using a ‘poke and patch’ approach with cream containing quercetin-loaded lipid microparticles followed by silicone-based MAP application, they achieved a 5.5-fold increase in intra-epidermal quercetin delivery compared to the control. Their study revealed quercetin mainly localised in the epidermis layer (approximately 2.23 µg/cm^2^) [44]. However, this amount was significantly lower than that achieved in our current work. Our formulation, integrating surfactants in the dissolving MAPs, successfully improved quercetin delivery to the epidermis layer, surpassing 1.5 mg of quercetin.

Quercetin is known for its diverse therapeutic activities, making it valuable for managing various diseases such as inflammation, cardiovascular disease, neurodegenerative disorders, cancer, ulcers, bacterial and viral infections, allergies, and respiratory conditions like asthma and hay fever [48]. When applied topically, quercetin exhibits potent antioxidant properties, shielding keratinocytes from external oxidative stressors and neutralizing free radicals [49]. This protective effect helps maintain endogenous antioxidant levels and prevents lipid peroxidation induced by UV exposure. Additionally, quercetin has demonstrated strong anti-inflammatory effects, surpassing other flavonoids in reducing inflammation induced by irritants [49]. The combination of its anti-inflammatory and antioxidant properties positions quercetin as a promising candidate for managing wound healing in particularly caused by diabetes mellitus [50]. To maximise this potential, an appropriate delivery vehicle is essential for successful skin delivery. Given that the oral bioavailability of quercetin is typically less than 10% [38], and there is limited research on its bioavailability through topical or transdermal delivery routes, utilising a MAP platform for quercetin delivery holds promise for enhancing local efficacy.

Regarding the proposed patch size, this can be extrapolated using the ex vivo skin deposition data and considering oral bioavailability, as there is limited available data for the transdermal pharmacokinetics of quercetin. To achieve an anti-inflammatory effect, at least 500 mg of quercetin per day is required to be dosed orally [51], with an oral bioavailability of less than 10% [38]. Accordingly, the dose of quercetin that needs to be effectively delivered is 50 mg. Therefore, correlating our ex vivo deposition data, which is able to deposit approximately 2 mg of quercetin and expected to be released sustainably over a period of time, with oral bioavailability, we can estimate the patch size for daily treatment in human adults to be 12.25 cm^2^. This patch size might be necessary to achieve systematic anti-inflammatory activity. Although the patch size in this study is relatively large compared to marketed transdermal patches, previous research has shown the successful application of large MAPs onto human skin. Moreover, it is important to note that this size is estimated to achieve systemic effect. Extensive studies are required to evaluate the dose needed to achieve anti-inflammatory activity locally, in which case smaller patches might be required to fulfill this purpose.

### Cytocompatibility studies

After evaluating both MAP formulations (F1 and F2) for their mechanical properties, insertion profile, and drug delivery efficiency, we opted for the MAP containing Soluplus^®^ (F2) for biocompatibility studies and in vitro anti-inflammatory assessment. This choice was due to the superior characteristics of the MAP F2 formulation, which boasted the highest drug loading, faster dissolution time, and greater drug delivery into the skin compared to F1. Previous reports have indicated that quercetin treatment significantly enhances the viability of HaCaT keratinocytes [52, 53]. To explore this further, we conducted MTT assays, live-dead staining, and proliferation assays to assess the cell viability, cytotoxicity, and proliferation of HaCaT cells following exposure to quercetin-loaded MAPs (F2) and blank MAPs.

**Fig. 8:**
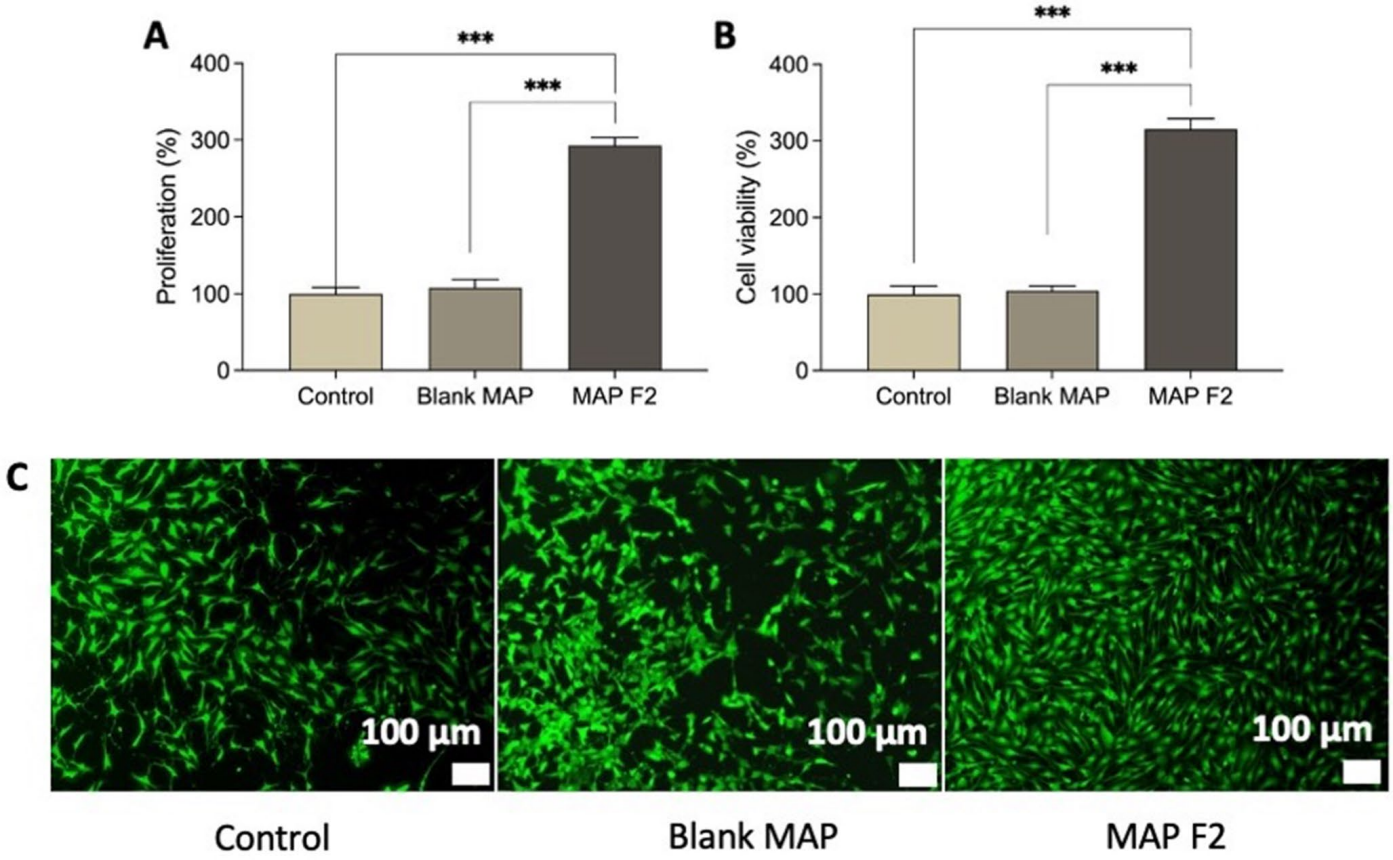
Biocompatibility assays on HaCat human cells. (**A**) MTT assay and (**C**) PicoGreen assay results showing total DNA content of cells on control, MAPS-Blank and, MAPS-quercetin (means + SD, *n* = 3). (**B**) Live/dead staining of HaCat cells on control (plate cells culture), MAPS-Blank and MAPS-quercetin, where green represents live cells and red represents dead cells

Our findings revealed no significant changes in the group treated with blank MAPs compared to the control, as shown in Fig. 8A. This was further supported by cell proliferation analysis (Fig. 8B) and the live-dead assays (Fig. 8C), indicating no notable alterations post-treatment with blank MAPs. In contrast, exposure to MAP F2 notably increased HaCaT cell count, depicted in Fig. 8A, a trend corroborated by the live-dead assay results (Fig. 8C). Additionally, the proliferation of HaCaT keratinocytes exhibited a significantly higher rate after treatment with MAP F2 (*p* < 0.05) (Fig. 8B). This observed stimulatory effect of MAPs loaded with quercetin on cell proliferation might be attributed to the increased incorporation of [3 H]thymidine into the cells, a phenomenon previously described in the HaCaT cell line [52]. Such results show potential in wound healing application, as during this process, more cell division is required to repair rapidly [52].

### In vitro anti-inflammation activity

**Fig. 9:**
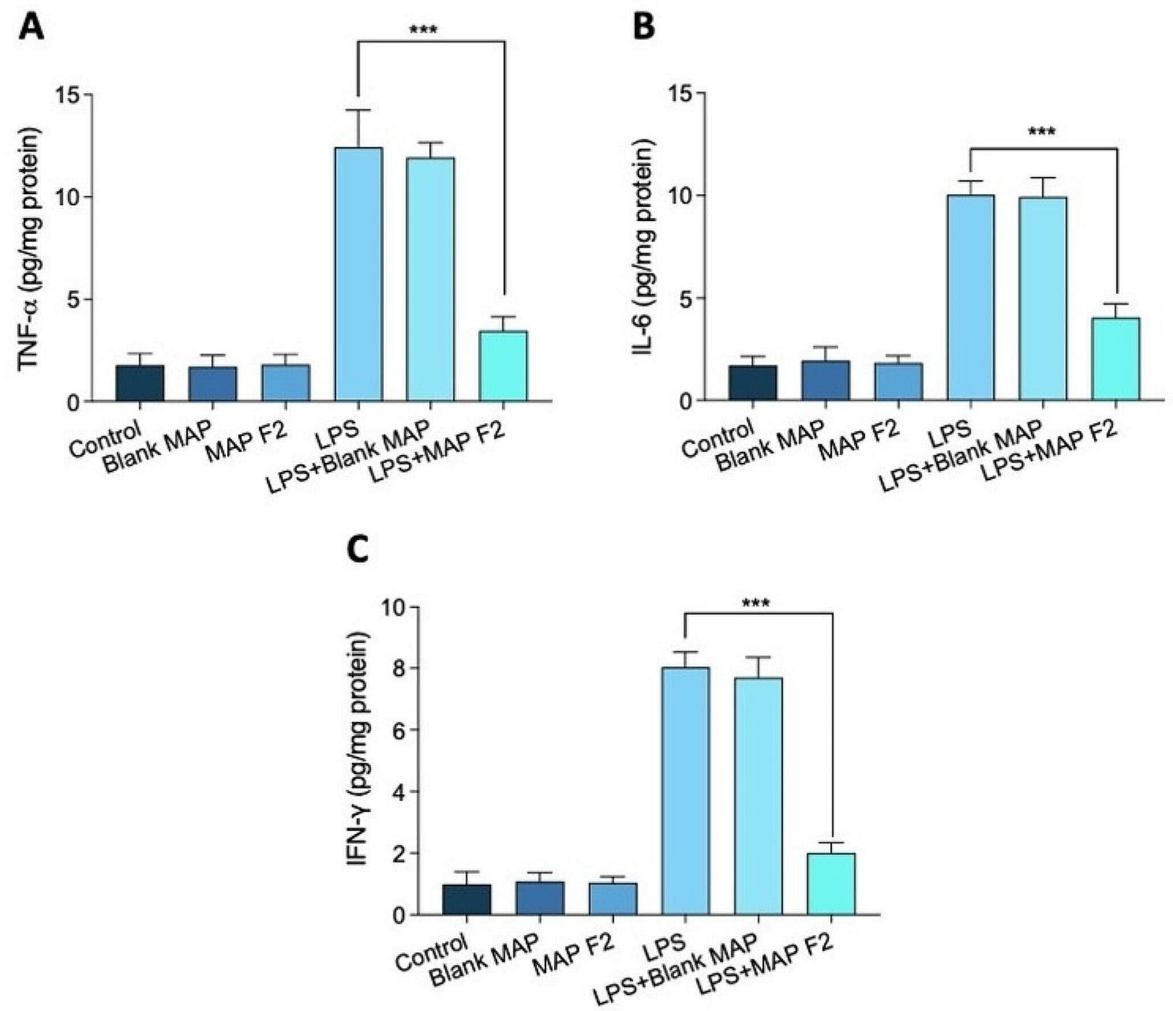
The changes in proinflammatory cytokine release were observed after treating human keratinocytes with LPS. This treatment led to increased production of tumour necrosis factor (TNF)-α, Interleukin 6 (IL-6), and Interferon-γ (IFN-γ), as determined by enzyme-linked immunosorbent assay in human keratinocytes. Subsequent treatment with MAPS-Quercetin resulted in decreased release of TNF-α, IL-6, and IFN-γ as shown in (**A**) TNF-α, (**B**) IL-6, and (**C**) IFN-γ (means + SD, *n* = 3)

In our study, we employed an LPS-induced inflammation model post-treatment with MAPs on keratinocyte cells to evaluate TNFα and INFγ production. Notably, LPS alone significantly increased (*p* < 0.05) cytokine production compared to the control group (Fig. 9). While treatment with MAP F2 showed a reduction in LPS-induced pro-inflammatory cytokines, these changes were not statistically significant (*p* > 0.05). Conversely, blank MAP treatment did not induce any alterations in inflammatory mediator production post LPS exposure. Importantly, in the absence of LPS treatment across the control, blank MAP, and MAP F2 groups, there was no increase in inflammatory mediator production, indicating the absence of inflammation in itself.

Quercetin treatments are recognised for their potent antioxidant and anti-inflammatory effects, as previously demonstrated in reducing both acute and chronic inflammation [54]. These treatments have also exhibited the promotion of cutaneous wound healing by enhancing fibroblast proliferation and migration while inhibiting inflammation, thereby accelerating wound closure and reducing oxidative stress. Studies have indicated that quercetin decreases TNF-α expressions [43]. Our results align with these findings, demonstrating high keratinocyte proliferation, good biocompatibility, and a noticeable reduction in pro-inflammatory cytokines in an in vitro inflammation model. These outcomes suggest that the MAP formulation with quercetin holds the potential to support wound healing or be employed to overcome inflammatory reactions.

Overall, the MAP loaded with quercetin fabricated in this study has demonstrated potential as an anti-inflammatory agent. However, the materials used in this study (PVA, PVP, and Soluplus^®^) need further assessment for safety and the possibility of accumulation in the body following short- and/or long-term application. While the polymers used in this study are biocompatible and do not cause toxicity to dermal cells, indicating that the fabricated MAP is safe for transdermal application, further studies are needed to investigate the polymer deposition profile resulting from dissolving MAP application before this technology is ready for clinical use.

## Conclusions

In summary, this study focuses on the fabrication, characterisation, and assessment of quercetin-loaded MAPs as potential alternatives for managing wound healing and inflammatory reactions. The MAPs developed in this research demonstrated robustness, withstanding pressures up to 32 N, akin to the pressure applied when administering them to the skin. Particularly, the inclusion of Soluplus^®^ as a surfactant resulted in high drug loading capacity, reaching up to 2.5 mg per patch, and complete dissolution within an hour of application on excised porcine skin. Skin deposition studies revealed the MAPs’ ability to deliver quercetin into various skin layers, achieving an impressive delivery efficiency of 80% over a 24-hour period. Moreover, the prototype MAPs displayed anti-inflammatory properties and proved to be biocompatible with human keratinocyte skin cells. Although these in vitro findings are promising, further investigations are imperative to assess efficacy in in vivo models, stability under different conditions with variations in temperature and humidity, and safety to evaluate polymer deposition before considering clinical application. Such experiments will be crucial to ensuring this system can have a meaningful clinical impact.

## Data Availability

The datasets generated during and/or analysed during the current study are available from the corresponding author on reasonable request.
